# Bright CsPbBr_3_ Perovskite Nanocrystals with Improved Stability by In-Situ Zn-Doping

**DOI:** 10.3390/nano12050759

**Published:** 2022-02-24

**Authors:** Yong-Tang Zeng, Zhan-Rong Li, Sheng-Po Chang, Arjun Ansay, Zi-Hao Wang, Chun-Yuan Huang

**Affiliations:** 1 Department of Applied Science, National Taitung University, Taitung 950, Taiwan; bryan27014156@gmail.com (Y.-T.Z.); year5340801@gmail.com (Z.-R.L.); 2 Advanced Optoelectronic Technology Center, Academy of Innovative Semiconductor and Sustainable Manufacturing, Department of Electrical Engineering, Institute of Microelectronics, National Cheng Kung University, Tainan 70101, Taiwan; changsp@mail.ncku.edu.tw; 3 Integrated Research and Training Center, Technological University of the Philippines, Manila 1004, Philippines; arjun_ansay@tup.edu.ph; 4 Green Energy Technology Research Center, Kun Shan University, Yongkang 710, Taiwan

**Keywords:** all inorganic lead halide perovskites, nanocrystals, Zn-doping, water-resistance stability, thermal stability

## Abstract

In this study, facile synthesis, characterization, and stability tests of highly luminescent Zn-doped CsPbBr_3_ perovskite nanocrystals (NCs) were demonstrated. The doping procedure was performed via partial replacement of PbBr_2_ with ZnBr_2_ in the precursor solution. Via Zn-doping, the photoluminescence quantum yield (PLQY) of the NCs was increased from 41.3% to 82.9%, with a blue-shifted peak at 503.7 nm and narrower spectral width of 18.7 nm which was consistent with the highly uniform size distribution of NCs observed from the TEM image. In the water-resistance stability test, the doped NCs exhibited an extended period-over four days until complete decomposition, under the harsh circumstances of hexane-ethanol-water mixing solution. The Zn-doped NC film maintained its 94% photoluminescence (PL) intensity after undergoing a heating/cooling cycle, surpassing the un-doped NC film with only 67% PL remaining. Based on our demonstrations, the in-situ Zn-doping procedure for the synthesis of CsPbBr_3_ NCs could be a promising strategy toward robust and PL-efficient nanomaterial to pave the way for realizing practical optoelectronic devices.

## 1. Introduction

Motivated by their promising applications in optoelectronics, synthesis techniques of organic-inorganic hybrid and all-inorganic lead halide perovskites (AILHPs) in forms of powder and nanocrystals (NCs) with improved characteristics have gained remarkable developments in the last decade [1,2,3,4,5,6,7,8]. Despite those astonishing breakthroughs, e.g., the ultrahigh power conversion efficiency in solar cells and theoretically high external quantum efficiency in perovskite light-emitting diodes (PeLEDs) [2,3], the transition of halide perovskite devices from the laboratory into commercial products has been greatly hindered by their poor stabilities and phase transition/deformation tendencies [9,10]. Actually, three-dimensional (3D) AILHPs (CsPbX_3_) show higher thermal decomposition stability for the inorganic nature when compared to their hybrid counterpart [10]. As a result, 3D AILHPs and their low dimensional cousins, Cs_2_PbX_5_ and Cs_4_PbX_6_, have become the research forefront concerning future development. While much research is aimed at device performance and novel applications of AILHPs, few tend to address the materials’ stability and toxicity issues. For instance, quaternary alkylammoniums such as tetraoctylammonium bromide (TOAB) and di-dodecyl dimethyl ammonium bromide (DDAB) were introduced in-situ or post-synthesis to partially replace the ligand of fatty acids to achieve higher dispersion stability [7,8]. Alternatively, Chen et al. introduced two-dimensional Bi_2_OS_2_ nanosheets into the perovskite precursor solution to form Pb-S bonds and suppressed the uncoordinated Pb^2+^ ions trap states [11]. Yong et al. have doped nickel ions to achieve AILHP NCs with near-unity PLQY. In comparison, Vashishtha et al. have added ZnBr_2_ into the precursor solution for CsPbBr_3_ NCs synthesis by the modified ligand-assisted reprecipitation (LARP) at room temperature with an equal-molar dibenzo-21-crown-7 ether added, to facilitate the fabrication of efficient PeLEDs [12]. However, no information about the stability of metal-doped AILHP NCs was given in these reports. In the present study, the strong band edge emission, improved PLQY, and enhanced stabilities of dispersion and water-resistance of CsPbBr_3_ NCs were demonstrated by Zn^2+^ ion doping based on the modified two-precursor hot-injection method [13].

## 2. Materials and Methods

To synthesize the green-emitting CsPbBr_3_ NCs, 204 mg of CsCO_3_ powder (99.99%, Alfa Aesar, Haverhill, MA, USA) and 650 μL of oleic acid (OA, 98%, Sigma Aldrich, St. Louis, MO, USA) were dissolved in 10 mL of octadecene (ODE, Sigma Aldrich) by stirring at 120 °C under N_2_ for 1 h, to obtain the Cs-oleate (CsOA) stock solution in advance. Meanwhile, 138 mg of PbBr_2_ (99.998%, Sigma Aldrich) and 85 mg of ZnBr_2_ (99.9%, Alfa Aesar) were added to the 10 mL of ODE in a three-neck flask by stirring at 150 °C under N_2_ for 30 min. For comparison, 276 mg of PbBr_2_ were used to synthesize the un-doped NCs. Subsequently, 1.5 mL of OA and oleylamine (OLA, >90%, Acros Organics, Waltham, MA, USA) were introduced into the solution to observe the complete dissolution of PbBr_2_ and ZnBr_2_. After the additional stirring for 1 h, 0.8 mL of the CsOA solution was quickly injected into the precursor solution, which was placed in an ice water bath after reacting for 5 s.

To purify the colloidal CsPbBr_3_ NCs, different amounts of ethyl acetate (EtOAc, Acros Organics) as the anti-solvent were added to the NC solution. The mixture was then centrifuged at 1000 rpm for 10 min and the supernatant was discarded. Then, 3 mL of hexane was added to disperse the precipitation and the solution was subjected to centrifugation again to discard the precipitation. In the following discussion, we denoted the un-doped and Zn-doped NCs as CsPbBr_3_ and CsPbBr_3_:Zn NCs, respectively.

In material characterization, the transmission electron microscope (TEM) and scanning electron microscope (SEM) images were scanned by a JEOL TEM 2100F microscope and a Hitachi SU8000 field-emission SEM (FE-SEM), respectively. The absorption and PL spectra of the NCs were performed by the UV-VIS spectrometer (LKU-5200, LINKO, Templestowe, VIC, Australia) and the FluoroMax-4 (Horiba Jobin-Yvon, Palaiseau, France) spectrofluorometer, while the absolute PLQY was obtained by combining the spectrofluorometer and a Quanta-Phi integrating sphere. The atomic force microscope (AFM) images were scanned in tapping mode by using the Bruker Innova AFM. The time-resolved PL (TRPL) spectra were measured using a pulsed diode laser as an excitation source and a Hitachi F-7000 spectrophotometer.

## 3. Results and Discussion

**Figure 1:**
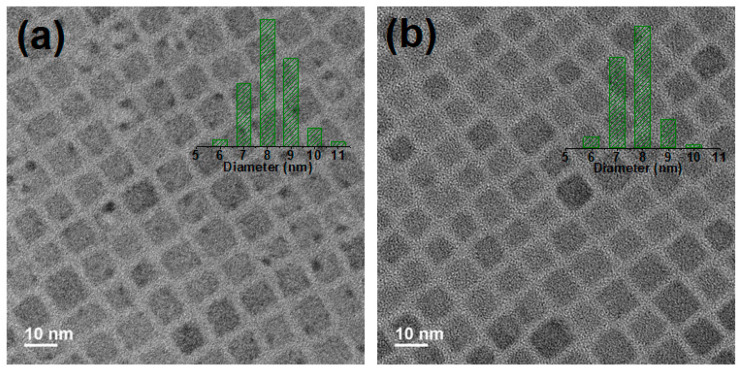
High-resolution TEM images of (**a**) CsPbBr_3_ and (**b**) CsPbBr_3_:Zn NCs. Insets show the histograms of edge lengths of corresponding NCs.

TEM and high-angle annular dark field scanning TEM (HAADF-STEM) images of the CsPbBr_3_ and CsPbBr_3_:Zn NCs are shown in Figure 1 and Figure S1 (see Supporting Information), respectively. Both of the doped and un-doped NCs exhibit cubic crystal structures, i.e., the Pm3m space group, and uniform size distribution with an average size of 8.1 ± 0.95 nm and 7.7 ± 0.84 nm, respectively. The average size of NCs is reduced by introducing the ZnBr_2_ for reaction, the size shrinking can be attributed to the reduction of the concentration of PbBr_2_ in the precursor solution for core lattice construction, whereas the ZnBr_2_, as the dopant, is only incorporated near the surface to compensate the structural defects. The small dark spots in Figure 1a are the product of electron-irradiation-induced decomposition, that is, the PbBr_2_ [14]. The decomposition of NCs further causes the Br desorption during TEM analysis [15]. Fewer dark spots in Figure 1b strongly suggest a higher strength of CsPbBr_3_:Zn NCs to resist the electron-irradiation induced decomposition.

**Figure 2:**
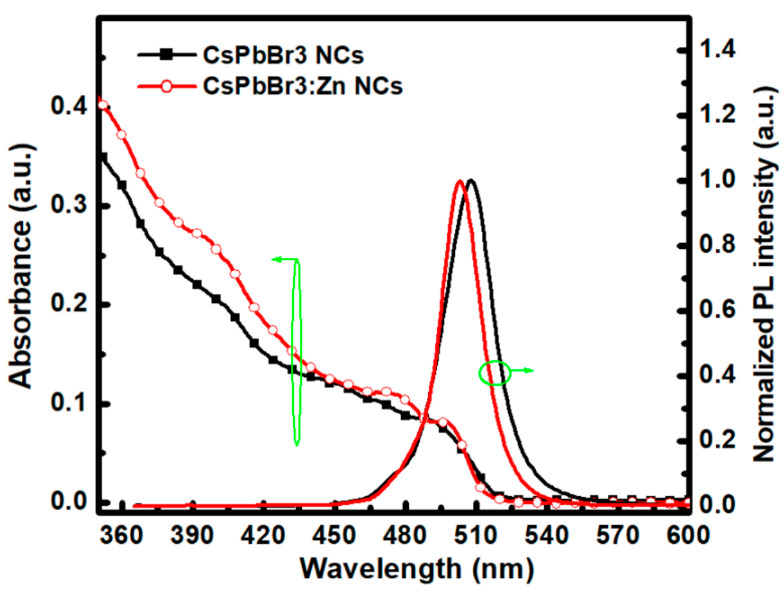
Absorption and PL spectra of CsPbBr_3_ and CsPbBr_3_:Zn NCs dispersed in hexane.

In Figure 2, the absorption and normalized PL spectra show that the first excitonic absorption peak/PL peak of the CsPbBr_3_ and CsPbBr_3_:Zn NCs are at 499 nm/507.2 nm and 495 nm/503.7 nm, while the full width at half maximum (FWHM) of the PL spectrum is 22.3 nm and 18.7 nm, respectively. There is no additional peak related to the dopant-induced defect levels, unlike that occurs in Mn^2+^-, Yb^3+^-, and Ce^3+^-doping studies [16,17]. The narrower FWHM and the more pronounced absorption peak suggest a more uniform size distribution for CsPbBr_3_:Zn NCs, or in other words, the higher color purity. Furthermore, the band gap energies are 4.216 eV and 4.236 eV, respectively, from the Tauc plot shown in Figure S2. It should be mentioned that different solvents including acetone (ACE), ethanol (EtOH), isopropyl alcohol (IPA), and EtOAc were respectively used to purify the CsPbBr_3_ NCs before the attempts of Zn-doping. From the comparison of absorption spectra shown in Figure S3, it was concluded that EtOAc was the best antisolvent to precipitate the maximum amount of NCs for higher production yield. This can be explained by the high polarity of ACE which can dissolve ionic perovskites whereas the protic nature of EtOH and IPA causes the NCs to be unstable in solvents [18]. From Figure 2, the concentration of the NCs in the stock solutions can be calculated based on Beer’s Law, *A* = ε*bc*, where *A* and *ε* are the absorbance and molar absorptivity at 400 nm, respectively. *b* and *c* are the 1 cm path length of a cuvette and the concentration of the solution under test. Taking the reported absorption cross-section at 400 nm for CsPbBr_3_ NCs into account [19], the molar concentration of the pristine CsPbBr_3_ and CsPbBr_3_:Zn NCs in hexane can be obtained as 1.23 × 10^−10^ mol/L and 2.43 × 10^−10^ mol/L, respectively. Obviously, the production yield (PY) of CsPbBr_3_:Zn NCs is about twice as high as that of CsPbBr_3_ NCs. Furthermore, the PLQY is increased from 41.3 to 82.9% after Zn-doping. It has been confirmed that the doping of divalent cations such as Ba^2+^, Sr^2+^, Sn^2+^, and Zn^2+^ tended to replace Pb^2+^ cations considering the charge balance and therefore stabilized the crystal phase [20]. In this regard, the observed enhancements in PY and PLQY can be attributed to the passivation of Zn ions into the Pb vacancy in NCs. Another distinct feature in Figure 2 is the blue-shift of absorption and PL peaks of CsPbBr_3_:Zn NCs. Considering the size shrinkage of CsPbBr_3_:Zn NCs, the blue-shift should be attributed to the enhancement of the quantum confinement effect.

**Figure 3:**
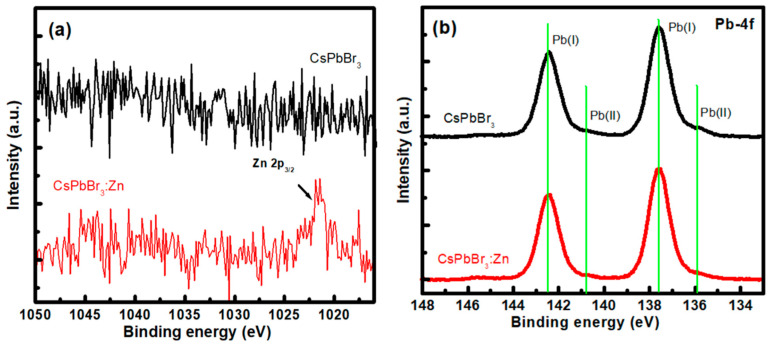
(**a**) Zn-2p and (**b**) Pb-4f core level XPS spectra of CsPbBr_3_ and CsPbBr_3_:Zn NCs deposited on Al-coated Si substrates.

High-resolution X-ray photoelectron spectroscopy (XPS) spectra of the samples are shown in Figure 3. For reference, the XPS survey scan spectra are compared in Figure S4. Despite the merely distinguishable signal, the XPS peak of Zn-2p_3/2_ appears at 1021.5 eV for CsPbBr_3_:Zn NCs in Figure 3a, indicating the evidenced incorporation of Zn ions and formation of bonding. Low Zn-2p_3/2_ peak intensity assures that Zn ions act as impurity-doping instead of being incorporated as the quaternary alloy compound CsPb_x_Zn_1-x_Br_3_. In Figure 3b of the Pb-4f spectra for both samples, two separated XPS peaks Pb(I) with binding energies of 138.5 eV and 143.2 eV correspond to the Pb 4f_7/2_ and 4f_5/2_ levels, which is well consistent with the Pb-Br bonding previously reported [21]. Two additional weak peaks Pb(II) appear at the shoulder should be related to the Pb-oleate [22], the by-product in the NC synthesis according to the following equation:

2Cs-oleate + 3PbBr_2_ **→** 2CsPbBr_3_ + Pb(oleate)_2_

The slight lower intensity of Pb(II) for CsPbBr_3_:Zn NCs indicates fewer residues of Pb-oleate after efficient antisolvent purification. Finally, it is worth mentioning that no obvious signal was detected in the energy range for metal Pb, which probably indicates that there are only trace amounts of Pb metals and the NCs are totally associated with the stoichiometric CsPbBr_3_.

**Figure 4:**
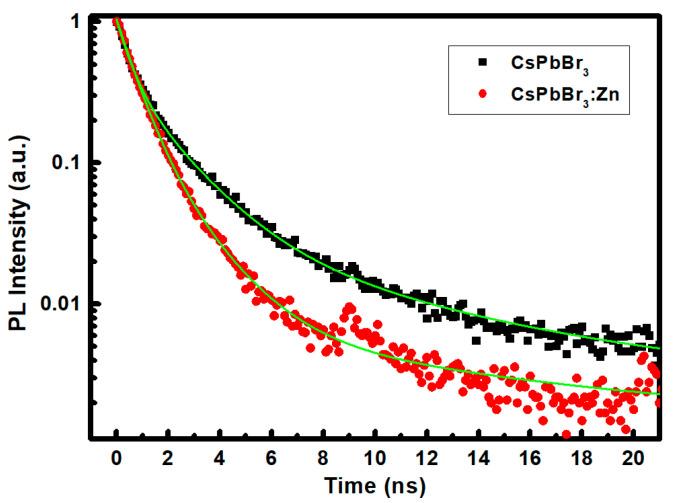
TRPL spectra of the CsPbBr_3_ and CsPbBr_3_:Zn NC solutions, respectively.

The TRPL decay curves are shown in Figure 4. To elucidate the mechanism responsible for the discrepancy, the exponential decay function was adopted to fit the decay curves. The bi-exponential decay model we previously used to fit the exciton relaxation within the Cs_4_PbBr_6_/CsPbBr_3_ nanocomposites does not represent the PL decay behavior of CsPbBr_3_ and CsPbBr_3_:Zn NCs. However, the triple-exponential function involved with trions and biexcitons can perfectly fit [23]. The triple-exponential function and the physical definition of each component are introduced in Support Information, and the fitted parameters are listed in Table S1. Obviously, the time constant for the recombination via the formation of trions and biexcitons is shortened from 1.79 ns and 0.68 ns to 1.67 ns and 0.54 ns, respectively, while both the generation probability and time constant for excitons are increased after incorporating the ZnBr into the precursor solution. The above attributions are responsible for the enhancement of the PLQY in CsPbBr_3_:Zn NCs. The shortening of the trion and biexciton recombination times explain the PL improvement of thiocyanate-treated CsPbBr_3_ quantum dots for better surface passivation [21].

**Figure 5:**
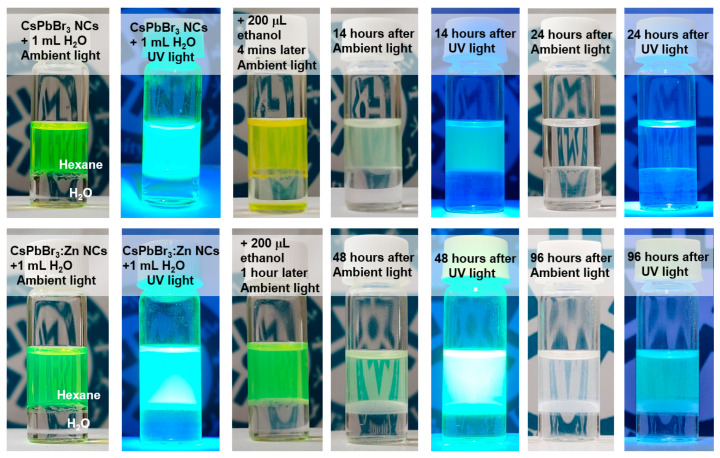
Photographs of variation of NC solutions with and without the ethanol addition under ambient light and UV light, respectively.

In evaluating the water-resistance ability of NCs, initially, 1 mL of water was added into the NC solutions with the same NC concentration. As shown in Figure 5, the NCs dispersed in hexane were not affected by the water addition due to the great difference in solvent polarity. As a result, no obvious decrease of the PL intensity from the NCs was observed for several days (not shown). To accelerate the hydrolysis reaction, 200 μL of ethanol were injected into each of the solutions, and then, as shown in the figure, the NCs turned to be yellowish within a few minutes, whereas the CsPbBr_3_:Zn NCs kept their green color. Both solutions appeared turbid because of decomposition (back to PbBr_2_ and CsBr) and aggregation of NCs [24]. Within one day, the decrease of concentration and PL intensity of CsPbBr_3_ NCs were clearly observed, and eventually, the solution with no NC dispersion became transparent again. The entire degradation process was significantly prolonged for CsPbBr_3_:Zn NCs to over four days. The precipitated white product, mainly the PbBr_2_ (because CsBr is highly dissolvable in water), accumulated at the solvent interface. From the above demonstration, it is evident that the surface passivation of Pb vacancies by Zn atoms is an excellent strategy to increase the water-resistance ability of AILHP NCs. Presumably, the Pb vacancy without surface ligands plays a role of breaking point for the water penetration and subsequent interaction between AILHP and water.

**Figure 6:**
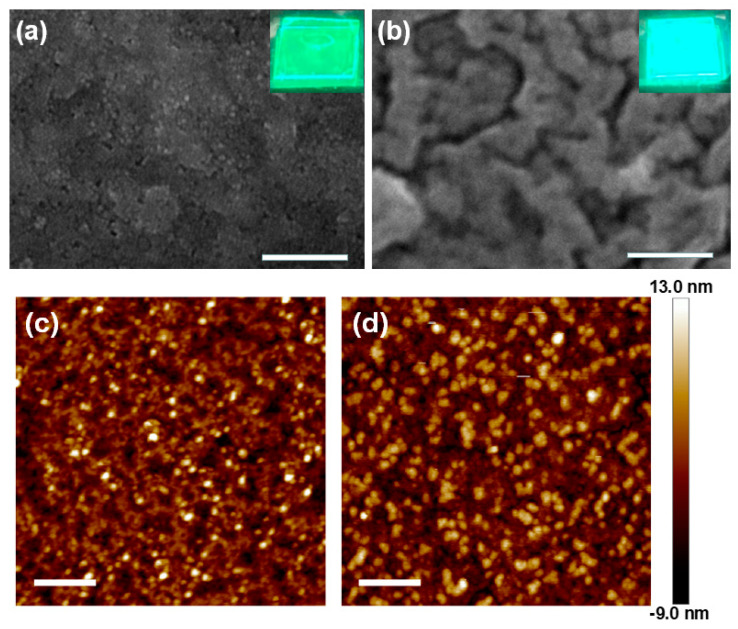
(**a**,**b**) SEM and (**c**,**d**) AFM images of the CsPbBr_3_ and CsPbBr_3_:Zn NCdeposited on glass, respectively. Insets of (**a**,**b**) are the corresponding top-view photographs under UV light illumination. The scale bar in SEM and AFM images represents 250 nm and 1 μm, respectively.

**Figure 7:**
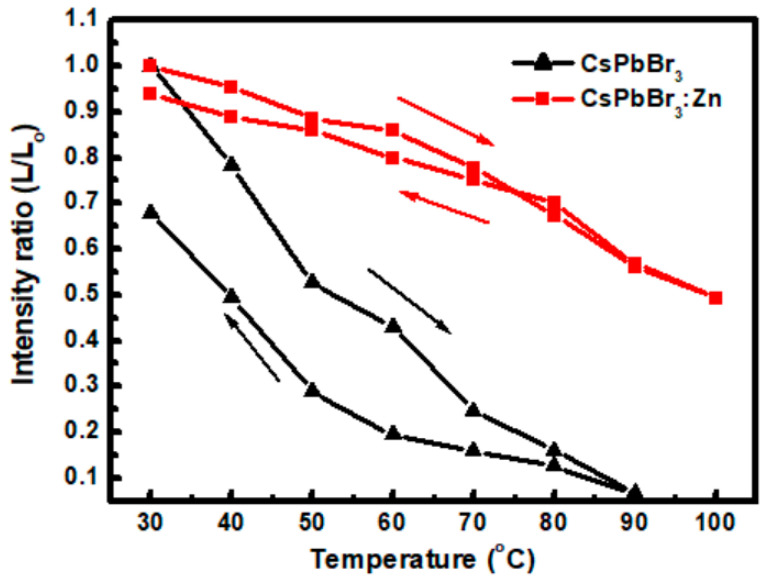
Variation of PL intensity ratio of NCs during the heating/cooling cycles. The excitation source is a UV-LED lamp.

In evaluating the process capability for electrically driven PeLEDs and the thermal stability, the CsPbBr_3_ and CsPbBr_3_:Zn NCs were spin-coated at 3000 rpm for 1 min onto glass substrates pre-cleaned by supersonication in detergent, ACE, and IPA sequentially, then UV-ozone treated for 25 min. From the SEM images of the coated NC films shown in Figure 6a,b, the close-packed CsPbBr_3_ NCs are observed, with plenty of voids that are not seen on the CsPbBr_3_:Zn NC film. However, the doped film exhibits enhanced grain boundaries, which may be due to the non-optimized NC concentration and spin speed. Both NC layers are continuous films. Inheriting the higher PLQY of CsPbBr_3_:Zn NCs, the doped NC film also shows a stronger PL intensity, as shown in the insets. In Figure 6c,d, the AFM images show that the surface roughness is 2.83 nm and 3.20 nm for CsPbBr_3_ and CsPbBr_3_:Zn NC films, respectively, suggesting that both of the films are smooth enough for PeLED fabrication as the forthcoming extension research. The thermal stability of NC films was characterized by analyzing the variation of PL intensity of films undergoing the heating and cooling cycles. To rule out the influence of oxygen and humidity, the stability test was carried out in a glove box. The results are shown in Figure 7. When the CsPbBr_3_:Zn NC film was heated to 100 °C, 49% of the original intensity was kept. In the cooling procedure, the PL intensity gradually increased and finally, 94% was recovered. By contrast, the un-doped NC film significantly lost 93% intensity at 90 °C and only 68% was recovered. Surprisingly, the thermal stability of CsPbBr_3_:Zn NCs is superior to that of the CsPbBr_3_ NCs embedded in the Cs_4_PbBr_6_ matrix we previously reported. In the present test, the maximum elevated temperature (90 °C and 100 °C) is above the reported temperature (88 °C) to induce the phase transition to the tetragonal phase [25]. Therefore, the thermal-induced degradation of PL intensity could be the result of strain and mechanical deformation which induce cracks and enlarge the grain boundaries for non-radiative exciton recombination. If this attribution is accurate, the procedure of Zn-doping should be beneficial to avoid strain accumulation and prevent structural deformation.

## 4. Conclusions

In conclusion, we demonstrated the outstanding characteristics and improved stabilities of CsPbBr_3_ NCs by in-situ Zn-doping. With the enhanced surface passivation, the Zn-doped NCs with an average size of 7.7 ± 0.84 nm revealed the twice higher PY (2.43 × 10^−10^ mol/L) and PLQY (82.9 %). The water-resistance ability of the doped NCs in the mixing solution of hexane, ethanol, and water showed a significantly extended period (over four days) until the complete decomposition. Moreover, the NCs could be spin-coated as a smooth and continuous film with a roughness of less than 3.5 nm for PeLED fabrication. Due to the increase in thermal stability of the Zn-doped NC film, 94% of the thermally-dependant PL intensity was retained. This is in contrast to the undoped film, which only retained 68% of the original PL intensity after a single heating/cooling cycle. The thermal stability of the Zn-doped NC film, in terms of the thermal-dependent PL intensity, preserved 94% of the original intensity, whereas the undoped film only remained 68% after one heating/cooling cycle. Based on our experimental results, the in-situ Zn-doping procedure can be a promising strategy for AILHP NCs toward efficient optoelectronic devices.

## Data Availability

Data presented in this article is available on request from the corre-sponding author.

## Supplementary Materials

The following supporting information can be downloaded at: https://www.mdpi.com/article/10.3390/nano12050759/s1, Figure S1: HAADF-STEM images; Figure S2: Tauc plot; Figure S3: Absorption spectra; Figure S4: Full XPS survey scan spectra; Table S1: Parameters for the bi-exponential decay fitting of the TRPL spectra.
