# Supplementary material for: Bright CsPbBr3 Perovskite Nanocrystals with Improved Stability by In-Situ Zn-Doping
Source: Nanomaterials (Basel). 2022 Feb 24;12(5):759. doi: 10.3390/nano12050759 (PMC8912077; doi:10.3390/nano12050759)
Supplement: Supplementary file 1 [file nanomaterials-12-00759-s001.zip › nanomaterials-1609988-supplementary.pdf]

Supporting Information

Bright $CsPbBr_3$ perovskite nanocrystals with improved stability by in-situ Zn-doping

Yong-Tang Zeng[1], Zhan-Rong Li[1], Sheng-Po Chang[2], Arjun Ansay[3], Zi-Hao Wang[2,4,*], and Chun-Yuan Huang[1,*]

[1] Department of Applied Science, National Taitung University, Taitung 950, Taiwan
[2] Department of Electrical Engineering, Institute of Microelectronics, Advanced Optoelectronic Technology Center, Academy of Innovative Semiconductor and Sustainable Manufacturing, National Cheng Kung University, Tainan 70101, Taiwan
[3] Integrated Research and Training Center, Technological University of the Philippines, Manila 1004, Philippines
[4] Green Energy Technology Research Center, Kun Shan University, Yongkang 710, Taiwan
* Correspondence: 10801013@gs.ncku.edu.tw (Z.-H.W); laputa@nttu.edu.tw (C.-Y.H.)

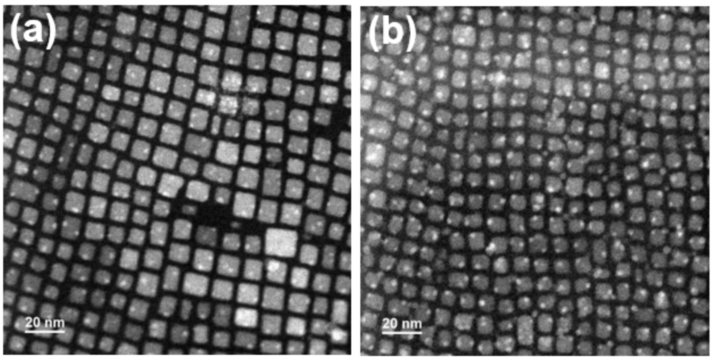

Figure S1. HAADF-STEM images of (a) $CsPbBr_3$ and (b) $CsPbBr_3$:Zn NCs.

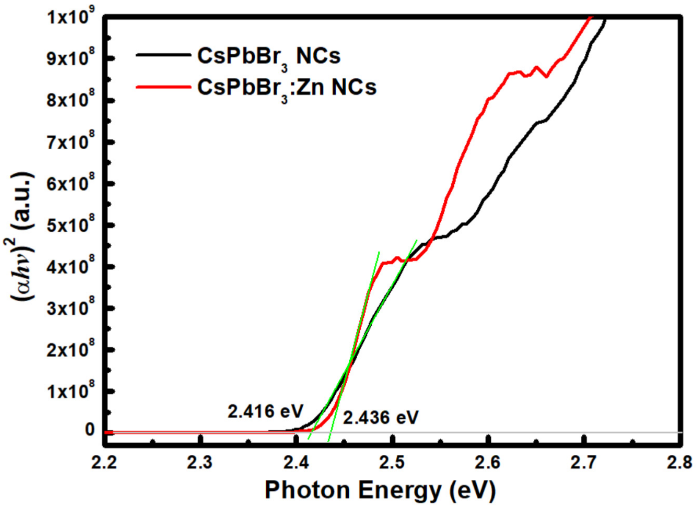

Figure S2. Tauc plot of $CsPbBr_3$ and $CsPbBr_3$:Zn NCs from their absorption spectra.

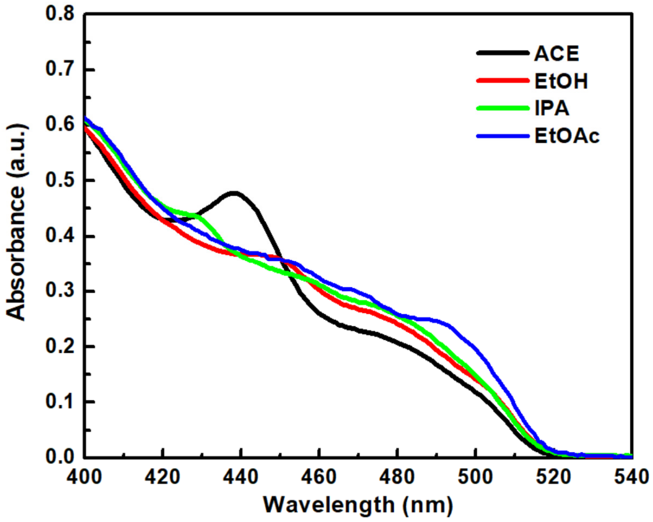

Figure S3. Absorption spectra of $CsPbBr_3$ NCs purified by different solvents.

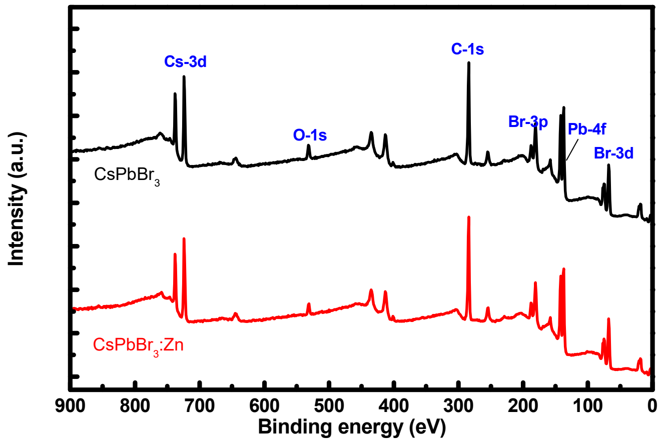

Figure S4. Full XPS survey scan spectra of $CsPbBr_3$ and $CsPbBr_3$:Zn NCs from their absorption spectra.

The PL decay curves of NCs were fitted by a triple-exponential decay model expressed as the following:

$$I(t) = I_0(A_x e^{-t/\tau_x} + A_{x*} e^{-t/\tau_{x*}} + A_{xx} e^{-t/\tau_{xx}}) \quad (1)$$

Where τ_x, τ_x, t_{xx} and A_x, A_{x*}, A_{xx} are the decay time constants of the exciton, trion, and biexciton and their relative generation probabilities, respectively. The average time constant can be calculated by:

$$\tau_{avg} = (A_x \tau_x^2 + A_{x*} \tau_{x*}^2 + A_{xx} \tau_{xx}^2)/(A_x \tau_x + A_{x*} \tau_{x*} + A_{xx} \tau_{xx}) \qquad (2)$$

The fitted parameters are shown in Table S1.

Table S1. Parameters for the bi-exponential decay fitting of the TRPL spectra.

Sample	A_x	τ_x (ns)	A_{x^*}	τ_{x^*} (ns)	A_{xx}	τ_{xx} (ns)	τ_{avg} (ns)
$CsPbBr_3$	0.04	7.33	0.37	1.79	0.59	0.68	2.66
$CsPbBr_3$:Zn	0.06	10.37	0.21	1.67	0.82	0.54	5.14
